# Analyzing the nexus between environmental sustainability and clean energy for the USA

**DOI:** 10.1007/s11356-024-32765-5

**Published:** 2024-03-22

**Authors:** Eyup Dogan, Kamel Si Mohammed, Zeeshan Khan, Rima H. Binsaeed

**Affiliations:** 1 Department of Economics, Abdullah Gul University, Kayseri, Turkey; 2 University of Ain Temouchent, Ain Temouchent, Algeria; 3 Faculty of Business, Curtin University, Miri, Malaysia; 4 Department of Management, King Saud University, Riyadh, Saudi Arabia

**Keywords:** COP27, Technological innovation, Clean energy, SDGS, Time series

## Abstract

Environmental sustainability is a key target to achieve sustainable development goals (SDGs). However, achieving these targets needs tools to pave the way for achieving SDGs and COP28 targets. Therefore, the primary objective of the present study is to examine the significance of clean energy, research and development spending, technological innovation, income, and human capital in achieving environmental sustainability in the USA from 1990 to 2022. The study employed time series econometric methods to estimate the empirical results. The study confirmed the long-run cointegrating relationship among CO_2_ emissions, human capital, income, R&D, technological innovation, and clean energy. The results are statistically significant in the short run except for R&D expenditures. In the long run, the study found that income and human capital contribute to further aggravating the environment via increasing CO_2_ emissions. However, R&D expenditures, technological innovation, and clean energy help to promote environmental sustainability by limiting carbon emissions. The study recommends investment in technological innovation, clean energy, and increasing R&D expenditures to achieve environmental sustainability in the USA.

## Introduction

The significance of environmental preservation and sustainability has reached unprecedented prominence. The escalating demand for natural resources imposes considerable pressure on ecosystems, giving rise to a diverse array of ecological issues such as atypical climate patterns, degradation of soil health, water source pollution, atmospheric contamination, biodiversity decline, and the impending risk of global warming. (Azam et al. 2023; Danish et al. 2023; Si Mohammed & Ugur Korkut 2023). Various international and national entities have implemented measures that incentivize green energy research and development expenditure and human capital. Building on the inherent benefits of green energy in helping promote economic growth and lower carbon dioxide emissions. The focus is now shifting to combining and amplifying multiple green energy sources. During a time marked by unprecedented population growth and escalating consumption levels, there has been a notable upsurge in the demand for sustainable energy sources.

The USA is recognized as one of the leading contributors to global carbon dioxide emissions, exerting a substantial influence on the overall levels of greenhouse gases in the atmosphere. The significant role played by this country emphasizes its influence on the dynamics of climate change and underscores the importance of its environmental policies and efforts to mitigate its effects. Between 1990 and 2022, the USA witnessed diverse carbon dioxide (CO_2_) emissions trends. The 1990s witnessed a notable upsurge attributed to economic expansion. The early 2000s witnessed a noteworthy culmination, driven by policy impact, energy dynamics shifts, and concerted endeavors toward enhancing efficiency. The increasing prominence of natural gas during the 2010s as a substitute for coal resulted in a further decline in emissions. Despite the prevailing policy concerns during the 2020s, the nation’s carbon dioxide (CO_2_) profile was still influenced by technological advancements and a growing shift towards green energy sources.

There has been a discernible surge in adopting and implementing green energy sources in the last few years. In various economic sectors, including road transportation and industry. Despite persisting challenges, this trend reflects the ongoing efforts to incorporate sustainable energy sources into these sectors (Dogan, Luni, et al. 2023a, b). However, there was a notable increase in the worldwide utilization of renewable energy, primarily attributed to the integration of wind and solar energy initiatives. According to Akram et al. (2020), Ansari (2022), and Dogan et al. (2023a), it is crucial to adopt and expand green energy sources to promote economic advancement and maintain ecological equilibrium. According to the International Energy Agency (IEA 2023), there has been a noticeable transition towards renewable energy, with a significant 13% global capacity growth in 2022. The increase in the utilization of green energy can be primarily attributed to the successful implementation of solar photovoltaic (PV) and wind projects and the prioritization of green energy sources over conventional forms of energy (Guan et al. 2023). Overall, the theory that links environmental sustainability with REC underscores the vital position accessible strength sources play in keeping our planet’s fitness and ensuring a sustainable future. This concept asserts that moving from fossil fuels to renewable energy is vital for mitigating environmental degradation. By changing the burning of fossil fuels with clean power resources like sun, wind, hydroelectric, and nuclear electricity, we will considerably lessen greenhouse gasoline emissions accountable for weather exchange. Furthermore, RE technologies promote energy efficiency, reduce air and water pollutants, and lessen our dependence on finite fossil gasoline resources. This transition not only fosters progressed air and water first-class but additionally helps sustainable growth, creates jobs, enhances strength protection, and contributes to the upkeep of biodiversity. Ultimately, mixing smooth electricity aligns with international sustainability desires and represents a pivotal step toward a more environmentally sustainable and resilient future.

The R&D investments facilitate advancements in energy storage, energy efficiency, and the utilization of alternative energy sources, consequently facilitating the gradual shift away from reliance on fossil fuels (Khan et al. 2023). Human capital development continues to be of utmost importance in tandem with these endeavors. Educational endeavors, training schemes, and the establishment of job prospects within the environmentally friendly industry play a crucial role in equipping the labor force with the necessary expertise and understanding to navigate and advance the realm of sustainable energy(Dogan & Pata 2022).

International and national entities are establishing the necessary groundwork for a sustainable future with reduced CO_2_ emissions. This is achieved by creating a favorable environment for adopting green energy, promoting technological innovation through research and development, and cultivating a skilled and knowledgeable workforce. Furthermore, the USA has a long patent development and technological innovation history. The USA saw significant technological innovation and patent activity from 1990 to 2022. Tech giants Microsoft and Apple led the 1990s tech boom. The 2000s saw biotechnology, software, and Google and Amazon breakthroughs. AI, renewable energy, and advanced computing were patent hotspots in the 2010s and 2020s. While patent quality and software patentability issues persisted, reforms like the America Invents Act of 2011 streamlined patent processes.

Our primary goal is to investigate whether human capital, green energy, and R&D will effectively reduce CO_2_ emissions for the USA from 1990 to 2022. Upon thorough analysis of existing scholarly works, we confidently affirm the pioneering character of this research paper, supported by multiple underlying principles. The primary contribution of this paper lies in its revelation of the significant role played by knowledge creation and innovation in the context of green energy development. Specifically, the paper emphasizes the crucial need for extensive improvements and integration of research and development activities, as well as the cultivation of human capital, with the ultimate goal of strengthening green energy initiatives and effectively mitigating CO_2_ emissions within the USA. Nevertheless, prior research has failed to acknowledge the conspicuous influence of promoting knowledge and innovation via dedicated R&D and human capital, particularly in mitigating emissions.

Moreover, the USA is trying to align with the principles outlined in the Paris Climate Agreement through its commitment to green energy and the augmentation of financial resources allocated to research and development endeavors. President Biden’s administration has created a $2 trillion concept to promote renewable electricity, infrastructure development, and various climate-focused tasks to achieve internet-0 emissions by 205 (Yuan et al. 2022). This bold plan includes significant investment in renewable energy sources, advancement in the electric car era, research and development initiatives, support for green patents, and funding for human enhancement, all essential components of this strategy. The research above fails to provide any discernible understanding of this particular facet. To rectify these research limitations, we have integrated two crucial factors: the allocation of resources towards research, development, and demonstration (RD&D) funding and the investment in human capital focused on advancing green energy technologies to mitigate the carbon dioxide emissions of the leading global emitter. We propose that Washington’s policy toward the USA’s green growth and green energy development needs to be ramped up to cater to the United Nations’ sustainability goals and attain the USA’s carbon neutrality target by 2050.

Furthermore, our study contributes simultaneously to policy discussions regarding attaining the SDGs. Green investments can facilitate resilient infrastructure development, accelerating progress toward SDG 9. Furthermore, clean energy investments focus on SDG 7, which can combat climate adversities. Such steps address SDG 13 on climate concerns. This paper’s structure will continue as follows. In “Literature review,” we present the review literature. The model, data, variables, and modeling strategy are described in depth in “Methodology.” The empirical results and robustness tests are discussed in “Main findings and discussions.” The policy implications are discussed in the final section of the study.

## Literature review

### The effect of green energy and CO_2_ emissions

Extensive scholarly research has extensively examined the consumption of renewable energy and its subsequent effects on mitigating environmental imbalances, particularly those caused by CO_2_ emissions (Ahmed et al. 2021; Si-Mohammed et al. 2022; Tiwari et al. 2023). The primary motivation for engaging in an academic inquiry of this nature arises from the expectation that fossil fuels will become obsolete by the conclusion of the current century (Voumik et al. 2023). The prevailing focus of scholarly investigations in this field highlights that adopting green energy consumption promotes environmental integrity, particularly in countries with strong implementation (Cruz-Soto et al. 2022; Rubio et al. 2023). An in-depth investigation was conducted by Abbasi and Adedoyin (2021), thoroughly analyzing GE’s implications on CO_2_ emissions. The study employed the historical emissions trajectory of the European Union (EU) as a benchmark for comparison. Their research revealed a noteworthy decrease in emissions levels within the examined countries, which can be attributed to using GE.

Similarly, a study by Wang et al. (2021) analyzed the impact of green energy consumption GE on CO_2_ emissions in specific regions along the Belt and Road (Fang et al. 2022), MENA (Lu et al. 2023), and AFRICA (Nathaniel et al. 2020). The proponents advocated for a prompt transition from reliance on fossil fuels in economic endeavors, employing the Generalized Divisia Index Method (GDIM) to support their findings. Additionally, a recent study conducted by Ehigiamusoe and Dogan (2022) provided a comprehensive understanding of the significant role that GE plays in restoring and preserving environmental sustainability in specific low-income nations.

### The effect R&D on CO_2_ emission

Several researchers have tackled the subject of R&D and have been introduced to limit the process of CO_2_ emission (Kihombo et al. 2021). The United Nations claims that climate change is the most pressing issue of our time and that humanity is at a crucial turning point. Investment in R&D plays a pivotal role in driving the progress of state-of-the-art technology and enhancing our comprehension of the surrounding world in China, notably, that launched low-carbon initiatives. Liu and Xu (2022) demonstrated that within China’s low-carbon pilot cities, various strategies, including innovation, green energy utilization, energy efficiency initiatives, and implementation of low-carbon transportation systems, have successfully mitigated CO_2_ emissions (Liu and Xu 2022). The study of Li and Ullah (2022) proposed implementing targeted measures. The researchers found a notable correlation between higher levels of scientific and technological innovation, as measured by research and development (R&D) expenditures and the number of green patents, and a substantial decrease in CO_2_ emissions within these urban regions. The research also revealed that various categories of low-carbon sectors exhibited distinct responses to innovations targeted at reducing CO_2_ emissions. Jiang et al. (2023) shows that from 1989 to 2021, investments directed towards research and development in environmental preservation and sustainable energy have significantly contributed to reducing carbon emissions in advanced economies. Moreover, a recent study in the USA (Kocak & Alnour 2022) examined a comparable association utilizing nonlinear estimation techniques. By employing the NARDL model and analyzing data from 1981 to 2020, the researchers emphasized the favorable effects of research and development (R&D) spending on upholding environmental sustainability in the USA. As a result, scholars have suggested increasing investment in the development of advanced knowledge as a means to revitalize the surplus of biocapacity in the USA. Similar findings corroborated in the developing and developed countries further the unidirectional causality from R&D to CO_2_ emission (Vitenu-Sackey & Acheampong 2022).

### The effect of human capital on CO_2_ emission

On the other hand, it is hypothesized that the growth of human capital has the potential to impact environmental conditions in various ways, both beneficial and detrimental. It is hypothesized that a nation with a higher proportion of educated individuals has a greater capacity to advocate for improved environmental quality from government entities, as education is linked to increased human capital reserves (Brasington & Hite 2005). Jahanger et al. (2023) documented that the development of human capital will contribute to the advancement of technological innovation, leading to more efficient energy utilization and decreased CO_2_ emissions. The research of Guloglu et al. (2023) focused on 26 OECD countries from 1980 to 2018. The study used a newly designed quantile common correlated effects mean group (QMG) model to investigate the impact of human capital, resources from nature, and the effect of green energy on environmental degradation. The study mainly concluded that human capital and clean energy sources favor the environment. Li and Ullah (2022) suggest that increased educational attainment has been associated with decreased carbon dioxide (CO_2_) emissions. In contrast, reduced academic progress has led to increased CO_2_ emissions over a prolonged period across the BRICS nations (1991–2019).

Using the ARDL approach, Pata and Ertugrul (2023) conducted a comparative study for this nation from 1988 to 2018, indicating that the human capital index contributes to preserving the environment.

### Gaps and scope in the literature

Based on the findings outlined above, we note the following gaps in the literature: (1) It is evident that the literature on how human capital and renewable energy affect CO_2_ emissions in different countries and regions is extensively rich. Although existing studies have documented how RE, R&D, and HC are interconnected, minor efforts have been made to link COP 27 and President Biden’s plan and 2025 agenda for top polluters like the USA. (2) Sustainable investment plays a crucial role in mitigating risks at the enterprise level and achieving long-term green energy objectives. Nevertheless, there appears to be a paucity of research within this specific field that examines the quantitative and breakpoint time series association between sustainability indicators, their determinants, and the green economy, particularly in the context of the United States. (2) Following specific regime shifts, the effectiveness of green technology applications and R&D in reducing carbon dioxide emissions becomes apparent. This underscores the necessity for further scholarly inquiry into the capability of green technologies and innovations to diminish carbon dioxide emissions. Green technology, investments in sustainability, and green energy efficiency are all critical determinants in predicting the levels of carbon dioxide emissions.

## Methodology

### ARDL approach

This study employs the autoregressive distributed lags (ARDL) approach (Pesaraned 2001) to examine the impact of renewable energy consumption on mitigating environmental imbalances, specifically those arising from CO_2_ emissions. The conclusions of our study are based on a combination of stationarity tests conducted at both the level and first difference. The ARDL model is suitable for analyzing time series with mixed levels of stationarity at 0 and 1. The ARDL model presents three distinct advantages for econometricians specializing in time series analysis. Firstly, the product exhibits remarkable flexibility, distinguishing itself from other models by its ability to accommodate variables regardless of their stationary characteristics. This implies that analysts are not obligated to establish stationarity as a prerequisite for modeling, thereby streamlining the initial analysis phases. Additionally, the model incorporates an inherent mechanism, referred to as the “bounds test,” to assess the presence of cointegration. This test aims to identify long-term equilibrium relationships between variables while avoiding complications related to individual integration orders. This allows for a more efficient evaluation of long-term associations. Finally, an important characteristic of the ARDL approach is its capacity to provide insights into both short-term dynamics and long-term relationships simultaneously. Incorporating a dual perspective is of great value, as it allows for a comprehensive comprehension of the interplay between variables in both temporary and enduring manners. This understanding is beneficial in facilitating informed decision-making across various domains, including policy formulation and financial prediction.

The equation can be rewritten in academic language as follows:

1 $$\Delta CO2={\beta }_{02}+{\delta }_{12}{GDP}_{t-1}+{\delta }_{22}{{\text{RECSN}}}_{t-1}+{\delta }_{32}{HCINDEX}_{t-1}+{\delta }_{42}{RDEVEXP}_{t-1}+{\delta }_{51}{TI}_{t-1}+\sum_{i=1}^{p}{\alpha }_{1i}\Delta {GDP}_{t-1}+\sum_{i=0}^{p}{\alpha }_{2i}\Delta {{\text{RECSN}}}_{t-1}+\sum_{i=0}^{p}{\alpha }_{3i}\Delta {HCINDEX}_{t-1}+\sum_{i=0}^{p}{\alpha }_{4i}\Delta {DEVEXP}_{t-1}+\sum_{i=0}^{p}{\alpha }_{5i}\Delta {TI}_{t-1}+{\varepsilon }_{1t}$$

The subsequent step in the process of estimating results involves assessing the number of temporal gaps in the model through the utilization of either the Akaike (AIC) (Akaike 1974), the Schwartz Bayesian (SBC) (Schwarz 1978), and Hannan–Quinn (HQ) (Hannan & Quinn 1979) information creations. The bounds test for cointegration is a statistical method used to determine whether a long-term relationship exists between two or more variables. It involves estimating an error correction model and conducting hypothesis tests to assess the presence of long run cointegrating association for the model.

The ARDL approach incorporates a bounds testing procedure to ascertain the presence of a cointegration relationship. The objective is to examine the collective significance of the coefficients associated with the lagged levels of the variables. Critical values are furnished for the *F*-statistics (or *t*-statistics), serving as boundaries. If the calculated test statistic exceeds the upper threshold, it is possible to reject the null hypothesis that there is no cointegration. If the value falls below the lower limit, it is impossible to draw any definitive conclusions. If the value of the test statistic lies within the specified bounds, the test results are considered inconclusive.

Following the identification of the duration of discrepancies, the subsequent phase entails the examination of the enduring integration association.

2 $${H}_{0}:{\delta }_{11}={\delta }_{21}={\delta }_{31}={\delta }_{41}={\delta }_{51}\dots$$

3 $${H}_{1}:{\delta }_{11}\ne {\delta }_{21}\ne {\delta }_{31}\ne {\delta }_{41}\ne {\delta }_{51}\dots$$

Subsequently, the Wald test evaluates the collective integration by comparing the measured *F* value against the critical *F* value. The H0 is rejected, and the H1 is accepted when the *F* value is found to be statistically insignificant, specifically when the *F* value is lower than the critical *F* value. The consistency of the ARDL model necessitates the absence of collinearity issues, which are not detected by the Durban-Watson DW coefficient. Utilizing the Lagrange Multiplier (LM version) is necessary in the literature. The lack of significance observed in the F value obtained (Breusch 1978) confirms the absence of collinearity.

Before illustrating the ARDL approach, the unit-root test is an essential statistical methodology used to ascertain the presence of a unit root in time series data. Identifying non-stationarity in a time series can be facilitated by detecting a unit root, as the presence of a unit root indicates non-stationarity. Thus, this test can be utilized to assess the stationarity of a series. Various statistical tests are available for conducting unit-root testing, including the structural break test. The augmented Dickey-Fuller structural break test is employed to examine the existence of a unit root. These checks are imperative to ensure a reliable and accurate time series data analysis.

### QR regressions

This study will use the QR to robust the QARDL results. Sim and Zhou (2015) proposed the panel QR in 2015 to analyze the independent influence of dependent variables in various market scenarios. Unlike standard regression models, which can only estimate the average effect and do not account for changing market conditions, QR can provide more robust findings by addressing difficulties, including heteroskedasticity, skewness, multicollinearity, and structural breaks (Cheng et al. 2023; Dawar et al. 2021; Mohammed & Mellit 2023).

### Data and model specification

This section provides econometric methods and model specifications for the study. In Eq. (4), the study provides the econometric specification of the model, where CO_2_ is carbon emission, GDP is economic growth, ${\text{RECSN}}$ is renewable energy, $HCINDEX$ represents capital human, while $DEVEXP$ is research and innovation technological TI, $\psi$_1_ to $\psi$_5_ are the coefficients. Finally, *ε*^*q*^_*t*_ represented the error term. Based on the prevailing findings in the literature above, the signs of economic growth ${\uppsi }_{1}={\text{GDP}}>0$. The respective following coefficients of the parameters renewable, capital human, and technological innovation (${\psi }_{2}, {\psi }_{3},\mathrm{ and} {\psi }_{5})$ will be negatively associated with CO_2_ emissions. Likewise, we envisage that green patents and the $RDEVEXP$ coefficient will negatively impact CO_2_ emissions.

The foundational framework for this research endeavor might be expressed in the following:

$${{\text{CO}}}_{2}\mathrm{ emissions }= f(\mathrm{Economic growth},\mathrm{ R}\&\mathrm{D expenditure},\mathrm{ Green energy},\mathrm{ and Humain capital},\mathrm{ technological innovation})$$

And expressed symbolically as in the following equation:

4 $${{\text{CO}}2}_{it}={\psi }_{0}+{\psi }_{1}{GDP}_{it}+{\psi }_{2}{{\text{RECSN}}}_{it}+{\psi }_{3}{HCINDEX}_{it}+ {\psi }_{4}{RDEVEXP}_{it}+ {\psi }_{5}{TI}_{it}+{\vartheta }_{it}$$

**Fig. 1:**
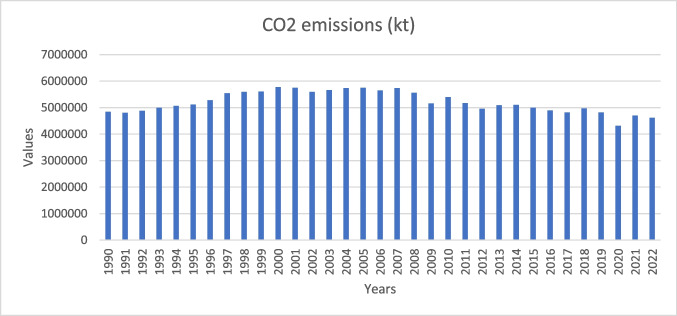
CO_2_ emissions in the USA (1990–2022)

**Fig. 2:**
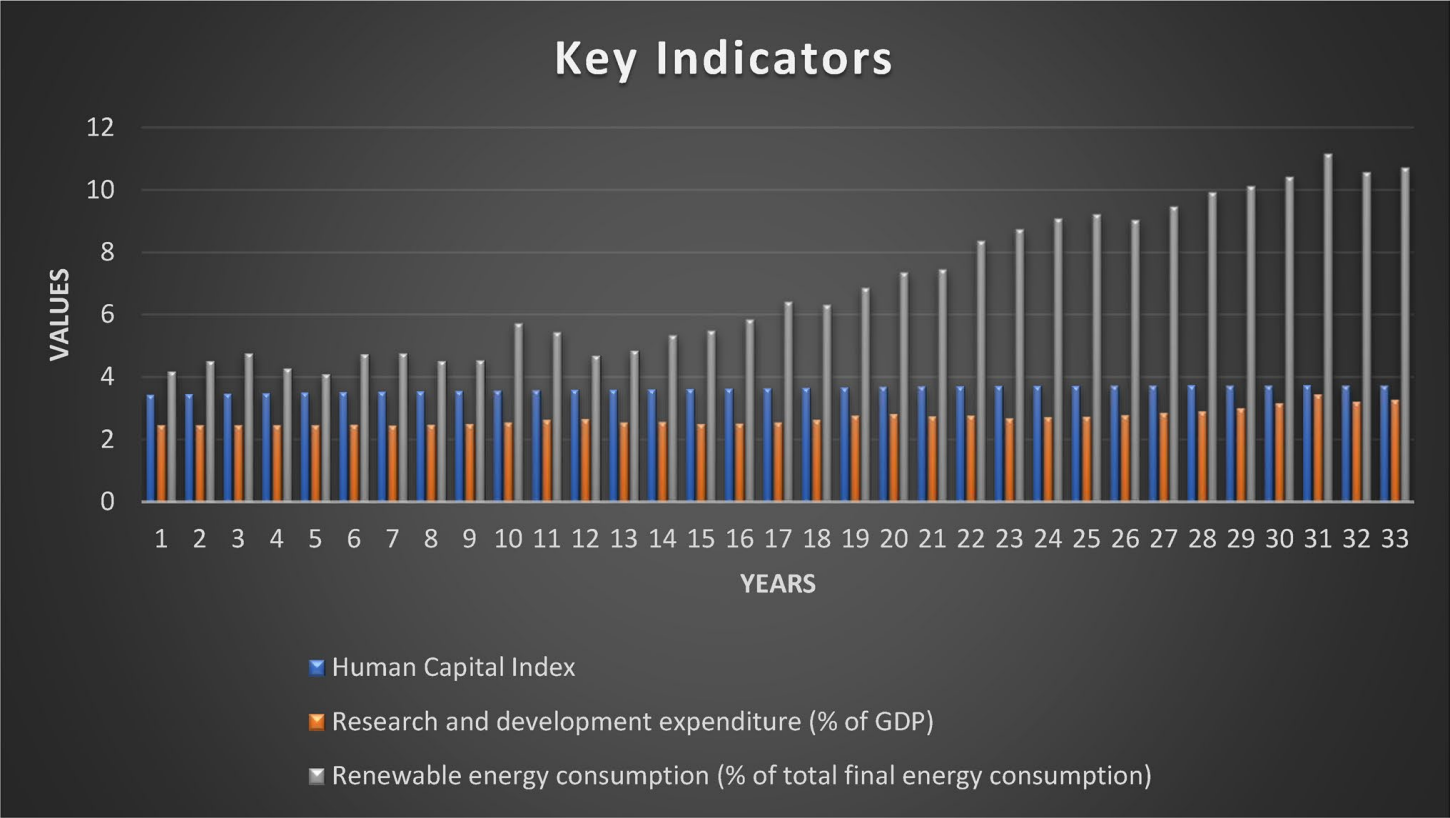
Trend data of human capital, R&D, and renewable energy

**Fig. 3:**
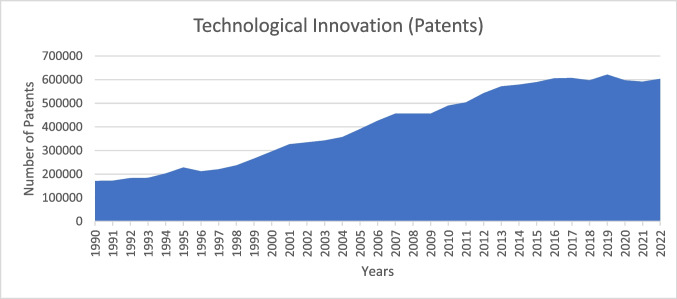
Technological innovation (1990–2022)

This study selected the USA to investigate the effect of GDP, green energy, R&D, and human capital on CO_2_ emissions. The sample period covers the years 1990 to 2020 on the parameters as presented in Table 1. Figures 1, 2, and 3 also show the data trend of CO_2_ emissions, innovation patents, and selective variables.

**Table 1:** Description of variables

Acronym	Data	Measurement unit	Source
CO_2_	CO_2_ emissions	k tons/year	WDI (2023)
GDP	GDP constant US 2015 dollar prices	%/year	WDI (2023)
${\text{HCINDEX}}$	Human capital index	Index	Penn World Table (2023)
RDEVEXP	Research and development expenditures	Percentage of GDP expenditure	WDI (2023)
RECSN	RECSN	RECSN	RECSN
TI	Technological innovation	Number of patents	OECD (2023)

## Main findings and discussions

This section will examine the initial findings derived from descriptive statistics analysis (Table 2). In addition to computing basic summary statistics, we also examine the presence of non-linearity, non-normality, skewness, and kurtosis in the selected series. The median values closely approximate their corresponding mean values and exhibit modest deviations, indicating a limited likelihood of outliers within the dataset. All designated time series exhibit positive values at their lowest points. Based on the standard deviation values, it can be observed that the data points tend to cluster around their respective mean values across all reports. The series of CO_2_ emissions, GDP, IT, and human capital exhibit a negative skew, indicating a greater concentration of values towards higher levels.

**Table 2:** Summary statistics

	CO_2_	GDP	HCINDEX	RECSN	DEVEXP	TI
Mean	5,210,821	1.53E + 13	3.629810	6.932795	2.703954	406,604.7
Median	5,117,037	1.60E + 13	3.642329	6.300000	2.631610	425,966.0
Maximum	5,775,807	2.10E + 13	3.738714	11.16000	3.450180	621,453.0
Minimum	4,320,533	9.80E + 12	3.435097	4.090000	2.450010	171,163.0
Std. Dev	397,791.0	3.34E + 12	0.097223	2.353379	0.260864	161,959.5
Skewness	− 0.093707	-0.124047	− 0.460575	0.400509	1.293448	− 0.094488
Kurtosis	1.990561	1.921531	1.911549	1.639173	3.934714	1.485534
Jarque–Bera	1.449374	1.683889	2.795710	3.428535	10.40287	3.202815
Probability	0.484476	0.430872	0.247126	0.180096	0.005509	0.201613

Conversely, the series of renewable energy and technological innovation display a positive skew, suggesting a higher concentration of values towards lower levels. The variables exhibit asymmetry, as evidenced by non-zero skewness coefficients. The kurtosis coefficients of the series indicate an excess kurtosis when the value surpasses three, indicating a deviation of these series from the normal distribution, except for CO_2_ and GDP. All the series have passed the Jarque–Bera test and demonstrated success in the normality test. This observation is apparent based on the probability values, which do not exhibit statistical significance at a 99% confidence interval, except for the research and development variable. This indicates that the series periods do indeed conform to a normal distribution.

The unit root test results for the selected data series are presented in Table 3. A stationarity test has been implemented to address the issues of cointegration and ARDL analysis. The ADF structural break unit root test, as proposed by Dickey and Fuller (1979), has been selected for our analysis, considering the existing literature. The ADF structural break test results indicate that the chosen series exhibits stationarity at the first difference for research and development. In contrast, the remaining variables are integrated at the first difference and conform to a normal distribution.

**Table 3:** Unit root test results

Tests	ADF **structural break**	Beak date
CO2	− 8.296(β)*	2011
GDP	− 6.913 (β)*	2009 (β)**
GE	− 9.019(_b_)**	2014
HC	− 9.56 6(_b_)*	2005 (_a_)*
R&D	− 5.411(_a_)*	2018
TINV	− 6.051 (_b_)*	1998

“***,” “**,” and “*” refer to the confidence interval at 99%, 95%, and 90% level, respectively

The results, presented in Table 4 and 5 below, are the cointegration test and creation information. Once the stationarity status of the chosen data has been verified, the subsequent step involves conducting the cointegration test. Three cointegration tests were employed to ascertain the presence of any long-term co-movement between carbon and its determinants. The bounds test for *F*- and *t*-statistics evaluates the enduring association among all-time series within the treatment cointegration ARDL model context. The results of this analysis sufficiently demonstrate the presence or absence of such a correlation. The *t*-statistic was determined to be 34.64, surpassing the lower and upper thresholds of the tabulated *F* and *t* values at significance levels of 10%, 5%, and 1%, with a value of 11.53. As a result, we successfully identified the presence of cointegration and long-term equilibrium between carbon dioxide (CO_2_) and the selected determinants of the variables in this study. The table displays the desired rank for the ARDL model, which was determined based on the outcomes of the applied methodology. The AIC, BIC, and HQ are commonly used statistical measures with respective weights of (1, 0, 0, 0, 0, 0). These criteria assess the relationship between CO_2_ emissions and their determinants***.***

**Table 4:** Results from the bounds test

*F*-bounds				
Test statistic	Value	Signif	I(0)	I(1)
*F*-statistic	34.61368	10%	2.75	3.79
		5%	3.12	4.25
		1%	3.93	5.23
*t*-bounds test				
*t*-statistic	− 11.53683	10%	-3.13	-4.21
		5%	-3.41	-4.52
		1%	-3.96	-5.13

**Table 5:** Selection criteria

Model	LogL	AIC*	BIC	HQ	Adj. *R*-sq	Specification
1	124.884267	− 7.305267	− 6.938833	− 7.183804	0.971658	(1, 0, 0, 0, 0, 0)

The results of the cointegration test confirm the viability of continuing with our final econometric modeling. As stated in the methodology section, the ARDL model was used to calculate the short- and long-run coefficients of the selected determinants. The results of the panel ARDL approach are shown in Table 6, as shown below. According to the study results, there is an evident correlation between a marginal rise of 1% in economic activity, as measured by the GDP, and a matching increase of around 1.58% in carbon dioxide (CO_2_) emissions in the near run. The long-term influence sees a relative drop of 1.3 units.

**Table 6:** Primary ARDL results

Variables	Coefficients	Std. error	Test statistics	*P*-value
**Short run coefficients**				
$\Delta$ GDP	1.584667	0.153643	10.31392	0.0000
$\Delta$ HCINDEX	4.767488	0.988259	4.824127	0.0001
$\Delta$ RECSN	− 0.104274	0.039610	− 2.632525	0.0146
$\Delta$ RDEVEXP DTI	− 0.110487	0.052522	− 2.103620	0.0461
$\Delta$ TI	− 0.025469	0.094487	− 0.269551	0.7898
ECM (− 1)	− 1.217216	0.105507	− 11.53683	0.0000
**Long run coefficients**				
GDP	1.301878	0.081910	15.89391	0.0000
HCINDEX	3.916714	0.707187	5.538442	0.0000
RECSN	− 0.085666	0.031727	− 2.700105	0.0125
RDEVEXP	− 0.090770	0.045440	− 1.997589	0.0572
TI	− 0.020924	0.078576	− 0.266290	0.7923

“***,” “**,” and “*” refer to the confidence interval at 99%, 95%, and 90% level, respectively. $\Delta$ is for short-run results.

In a similar vein, it can be seen that a mere 1% augmentation in human capital inside the USA results in a commensurate rise in carbon dioxide emissions of 4.7% and 3.9% throughout the medium and long term, respectively. Contrarily, a marginal rise of 1% in the adoption of renewable energy sources has shown a favorable influence on the state of the environment, leading to a decrease of 0.11 units of carbon dioxide (CO_2_) emissions in the immediate timeframe and 0.08 units in the extended timeframe. Likewise, R&D has a favorable influence by substantially mitigating carbon (CO_2_) emissions, achieving reductions of 0.011 and 0.9 in the immediate and extended timeframes, correspondingly. Concerning the influence of TI on (CO_2_) emissions, it is evident that a 1% augmentation in TI results in a decrease in CO_2_ emissions by 0.02% over both the short and long terms. Nevertheless, it is crucial to acknowledge that the observed impact lacks statistical significance.

The BG LM test is essential to confirm a model’s absence of autocorrelation problems. This examination encompasses two hypotheses: the null hypothesis posits the absence of autocorrelation, while the alternative hypothesis proposes the existence of autocorrelation. In the present scenario, the computed *F*-statistic is 1.29, surpassing the significance level of 5%. Nevertheless, the *F*-statistic does not exhibit statistical significance. As a result, the null hypothesis (H0) is accepted, leading to the conclusion that there is a lack of evidence supporting the presence of autocorrelation in the model. See Table 7. The robustness results are provided in Table 8.

**Table 7:** Diagnostic test (serial correlation)

*F*-statistic	1.285837	F(5,19)	0.3109
Obs * *R*-squared	8.090463	Chi-square (5)	0.1513

**Table 8:** Robustness (quantile regression)

	Coefficient	**Z**	*P*-value
*Q*_25th_			
GDP	0.465586	9.934459	0.0000
HCINDEX	2.105605	1.229506	0.2291
RDEVEXP	− 0.422756	− 2.777482	0.0097
RECSN	− 0.480466	− 5.911299	0.0000
TI	− 0.005281	− 0.059256	0.9532
*Q*_50th_			
GDP	0.473332	9.707038	0.0000
HCINDEX	2.073192	1.115960	0.2739
RDEVEXP	− 0.591466	− 3.480541	0.0017
RECSN	− 0.396547	− 4.495477	0.0001
TI	− 0.018498	− 0.183644	0.8556
*Q*_75th_			
GDP	0.469895	14.45442	0.0000
HCINDEX	2.190175	1.710278	0.0983
RDEVEXP	− 0.587780	− 3.783589	0.0007
RECSN	− 0.394043	− 5.815945	0.0000
TI	− 0.021525	− 0.297798	0.7681
*Q*_95th_			
GDP	0.468623	12.85645	0.0000
HCINDEX	2.518581	1.690099	0.1021
RDEVEXP	− 0.565794	− 3.809467	0.0007
RECSN	− 0.396351	− 5.782460	0.0000
TI	− 0.052540	− 0.634828	0.5307

**Fig. 4:**
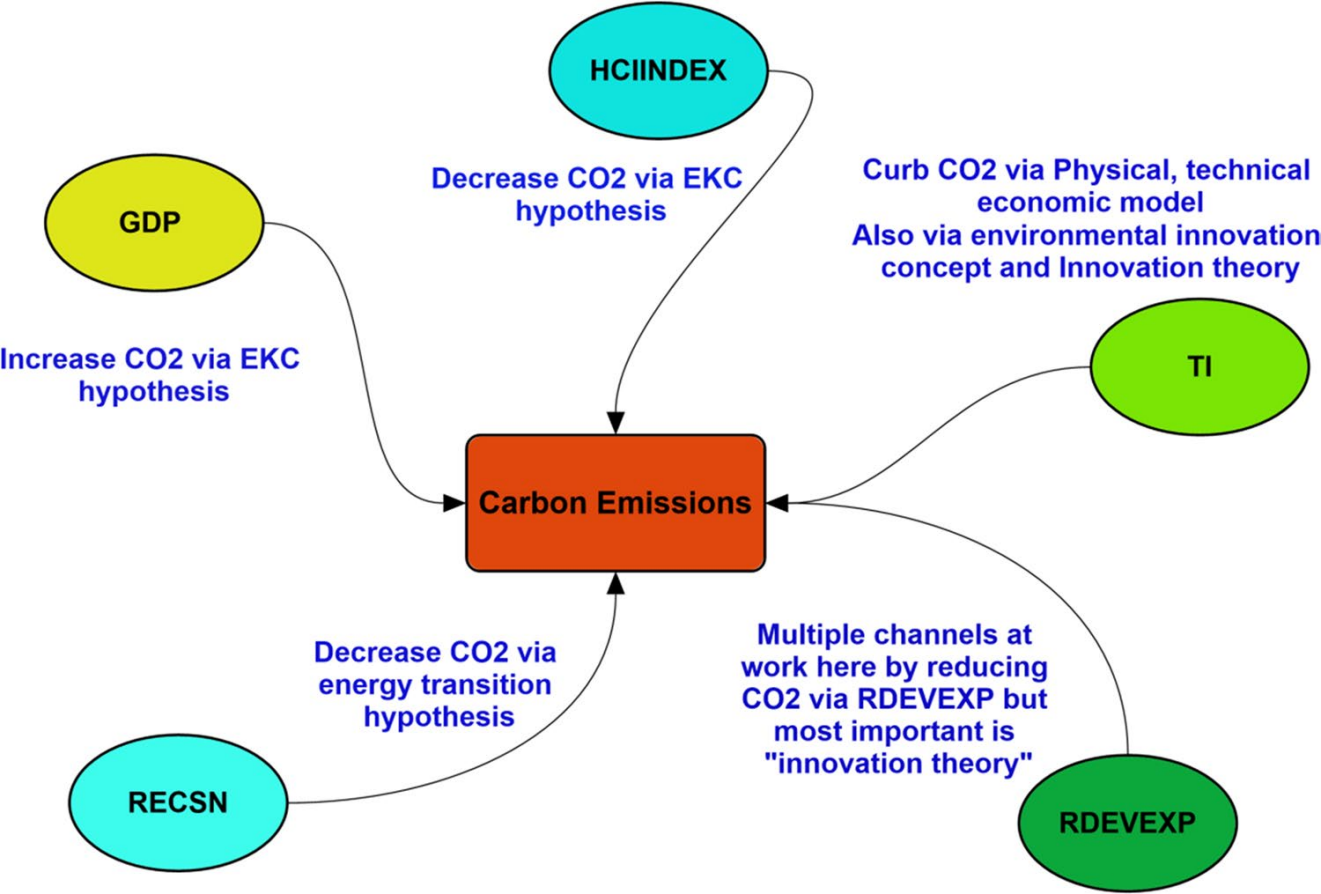
Theoretical framework flowchart

**Fig. 5:**
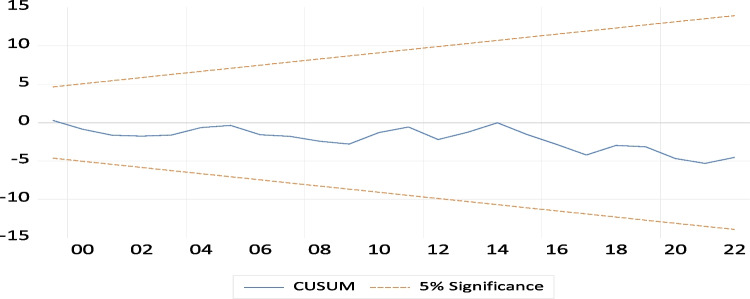
CUSUM results

**Fig. 6:**
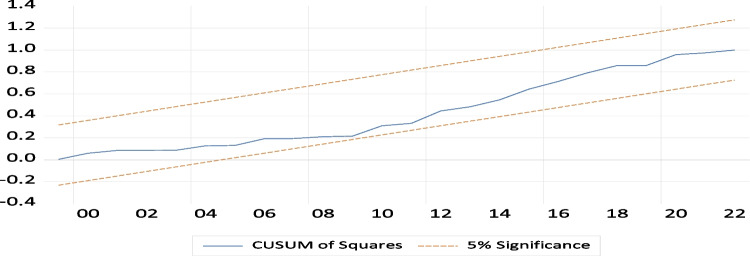
CUSUM SQUARE results

The primary aim of the CUSUM and CUSUM square tests is to evaluate the executed addition’s structural and dynamic stability. To assess the stability of the established model, we examine the depicted Fig. 4. The graph’s trend line falls within the critical boundaries, indicating statistical significance at a 5% level. This finding suggests that the coefficients of the various factors demonstrate stability, as evidenced by the CUSUM stability test via Figs. 5 and 6.

**Fig. 7:**
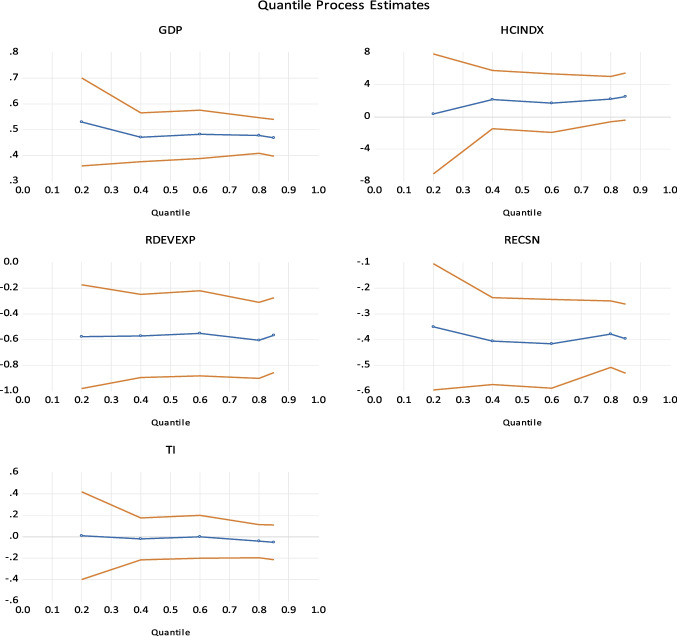
Quantile result coefficients graphically

The quantitative research results have been presented in a panel data format and are exhibited in Table 6. This study analyzes the influence of explanatory variables, namely, renewable energy, research and development (R&D), human capital, and technological innovation, on carbon dioxide (CO_2_) emissions within the USA. This study provides empirical evidence that aligns with the findings of the (ARDL analysis, which examines the impact of independent variables on various quantiles (25th, 50th, 75th, and 85th) of all variables. The results indicate that increased GDP and human capital in the USA produce higher CO_2_ emissions. Clean energy, R&D, and technological advancements help the immediate and long-term reduction of CO_2_ emissions. However, it is worth noting that the impact of TI is statistically insignificant across all quantile levels, and human capital only shows significance at the lower and medium quantiles. Figure 7 shows quantile regression coefficients graphically.

## Discussions

As mentioned above, the findings demonstrate a statistically significant and positive sign effect of (GDP) on short-term CO_2_ emissions. The findings of this study indicate that a marginal increase of 1% in economic activity is linked to an estimated increase of approximately 1.58% in CO_2_ emissions. The correlation between this relationship can be attributed to the predominant reliance on the combustion of fossil fuels for energy in the context of production processes, thereby leading to increased emissions. Furthermore, it is imperative to consider the temporal aspect of this effect. Over a prolonged period, the impact undergoes a decrease of 1.3, suggesting a gradual diminishing of its influence as time progresses. This phenomenon can be interpreted as a reduction in the scale of CO_2_ emissions per unit of economic productivity. This implies that while there is an initial increase in CO_2_ emissions due to economic growth, subsequent advancements and adaptations tend to mitigate this effect over an extended period. These findings are consistent with previous studies and indicate a positive relationship between GDP growth and CO_2_ emissions (Ahmed et al. 2021; Fakher et al. 2023; Si-Mohammed et al. 2022; Tiwari et al. 2023). When analyzing the correlation between human capital and CO_2_ emissions, exploring how an increase in human capital can impact economic activities, and consequently, the resulting environmental outcomes are valuable. Human capital, which encompasses the educational attainment, skill set, and overall health of a population, holds significant importance in shaping economic productivity and facilitating the development of innovative solutions. The data presented in Table 6 demonstrates that in the USA, an increase of 1% in human capital is associated with a significant increase in CO_2_ emissions of 4.7% and 3.9% in the short and long term, respectively.

The substantial growth can be analyzed from multiple viewpoints. First and foremost, the enhancement of human capital is anticipated to yield increased economic productivity and innovation. While this phenomenon enables the economy’s growth, it can also lead to heightened energy consumption and industrial activities, significantly contributing to CO_2_ release. The initial rise in emissions, which corresponds to a 4.7% increase, can be ascribed to the immediate impacts of economic expansion stemming from the augmentation of human capital. Over a prolonged period, there is a slight decline of 3.9% in the concurrent emissions increase. The decrease in relative figures can be attributed to implementing more efficient and environmentally sustainable technologies and methodologies, which are often associated with advancements in human resources. Populations exhibiting a notable degree of education and expertise tend to adopt and promote innovative strategies that improve energy efficiency and reduce carbon emissions, thereby mitigating the overall environmental footprint (Dogan & Pata 2022; Khan et al. 2023). Moreover, the findings above indicate that a slight augmentation of 1% in adopting renewable energy is correlated with a reduction of 0.11 units of CO_2_ emissions in the near term. The precipitous decrease can be ascribed to replacing energy derived from fossil fuels with more environmentally friendly and sustainable alternatives. Renewable energy technologies harness energy from sustainable sources, resulting in minimal or non-existent direct emissions throughout their operational lifespan. As a result, these technologies play a role in decreasing the carbon emissions associated with each unit of energy produced.

Over a prolonged duration, it has been observed that there is a slight decrease in CO_2_ emissions, specifically by 0.08 units, in direct correlation to a 1% increase in the adoption of green energy sources. Despite its seemingly contradictory nature, the apparent decrease in the emission reduction rate can be attributed to many factors. As economies undergo expansion and observe an escalation in energy requirements, there is a simultaneous augmentation in the aggregate quantity of renewable energy necessary to maintain or further reduce emissions (Cruz-Soto et al. 2022; Rubio et al. 2023). Moreover, the extensive adoption of renewable energy sources necessitates substantial investments in infrastructure and advancements in energy storage and grid management systems, potentially producing supplementary emissions (Dogan et al. 2022). Nevertheless, it is imperative to underscore renewable energy's cumulative and escalating benefits. While the immediate impact of directly substituting fossil fuels may be evident, the long-term benefits are multifaceted. The factors above encompass facilitating technological advancements, implementing improved renewable energy systems, and gradually phasing out energy sources that generate significant carbon emissions. The gradual decrease in carbon emissions within the energy sector, facilitated by the widespread adoption of renewable energy sources, will play a crucial role in tackling climate change and reducing the overall carbon footprint of human activities(Pata 2023; Shahzad et al. 2023). In analyzing the effect of the (R&D) and TI within the framework on environmental degradation, a multifaceted interplay of variables is observed to influence the CO_2_ emissions. The R&D facilitates and advances technological advancements. This entails efforts to develop innovative technologies, improve existing ones, and enhance overall efficiency, directly influencing the capacity to mitigate emissions.

The results indicate that engaging in R&D endeavors has a noticeable impact on reducing CO_2_ emissions. In the short term, there is an initial modest decrease of 0.011 units, which is succeeded by a more significant and persistent reduction of 0.9 units in the long term. The outcome variation can be attributed to the inherent attributes of research and development endeavors. In the short term, adopting incremental innovations and optimizations can generate prompt, albeit relatively modest, improvements in emission reduction. Over time, the cumulative effect of ongoing research and development efforts and the progress and widespread adoption of groundbreaking technologies significantly reduces carbon dioxide (CO_2_) emissions. These findings align with previous studies (Jiang et al. 2023; Vitenu-Sackey & Acheampong 2022). Finally, this statement highlights the importance of technological innovation (TI) in influencing CO_2_ emissions. A robust inverse relationship has been observed between a 1% augmentation in technological innovation (TI) and a 0.02% decline in carbon dioxide (CO_2_) emissions, persisting over immediate and extended periods. This demonstrates the long-lasting benefits of technological advancements in promoting the development of environmentally friendly and sustainable solutions in various sectors of the economy. However, it is crucial to consider the statistical significance of the correlation between TI and CO_2_ emissions when interpreting these findings. While indicating a potential reduction in emissions, the phenomenon under observation lacks statistical significance. Within the study’s parameters, the observed association between TI and emission reduction does not meet the criteria to establish a causal connection conclusively. The transmission mechanism linking renewable energy, green patents, and human capital to the reduction of CO_2_ emissions operates through a dynamic and interconnected system. Renewable energy directly supplants fossil fuel usage, significantly reducing emissions, while also influencing energy market dynamics, potentially making sustainable options more economically viable (Hu et al. 2022). Green patents catalyze this process by driving technological innovations that are more efficient and have smaller carbon footprints, extending their impact beyond the energy sector into wider economic realms. Human capital, with its focus on specialized knowledge and skills in green technology and environmental practices, is essential for the effective implementation and maintenance of these innovations. Additionally, a workforce educated in sustainability principles advocates for environmental policies and engages in behaviors that further reduce emissions. This integrated mechanism creates a reinforcing cycle: advancements in renewable energy technologies foster further innovation through green patents, and an educated, skilled workforce enhances the development and adoption of these technologies, collectively accelerating the reduction in CO_2_ emissions. Possible explanations for this phenomenon may encompass variability within the data, the impact of external factors, or the observed effect’s relatively negligible magnitude.

## Conclusions and policy recommendations

### Conclusions

Environmental sustainability is currently one of the key targets of many countries, especially post-sustainable development goals targets. Most recently, the Conference of Parties (COP27) further highlighted the importance of environmental sustainability. Therefore, in the context of the SDGs and COP27, the current study aimed to evaluate the impact of green energy, technological innovation, human capital, and R&D expenditures on carbon emissions in the USA for 1990–2022. The research used time series information sourced from the World Bank. The research demonstrated that human capital and income level favorably influenced CO_2_ emissions in the long term. This indicated that growing income levels and human capital might increase CO_2_ emissions in the long term. In contrast, the research found that rising R&D spending, technical innovation, and green energy help reduce CO_2_.

### Policy recommendations

The regulatory and policy framework of President Biden’s $2 trillion initiative on renewable energy and climate change is a critical component for ensuring the successful transition to a sustainable, low-emission economy. This framework necessitates the develop of new regulations and the revision of existing ones to mandate and incentivize renewable energy use, encompassing stricter energy efficiency standards and emissions guidelines. Key to this approach is the strategic deployment of financial incentives, such as tax credits and subsidies, to accelerate the adoption of green technologies by both producers and consumers. Public–private partnerships will also play a pivotal role, fostering collaboration and innovation in sustainable practices.

In addition to regulatory changes, this policy framework must focus on upgrading the national energy infrastructure to support a higher proportion of renewables, including modernizing the electric grid and developing infrastructure for electric vehicles. Equally important is the emphasis on climate resilience, ensuring that infrastructure and communities are prepared for the impacts of climate change. This framework also encompasses educational and workforce training initiatives, preparing a skilled labor force for the emerging green economy.

Effective monitoring and enforcement mechanisms are essential to ensure compliance with new standards, necessitating robust oversight systems. The USA must align its policies with international environmental standards and participate actively in global climate initiatives. Finally, the framework should address the social and economic impacts of the green transition, aiming to minimize adverse effects on communities dependent on traditional energy sectors and ensuring equitable access to renewable energy benefits. This comprehensive policy and regulatory approach is crucial for steering the country towards a more sustainable and environmentally responsible future. Moreover, the study highlights and emphasizes the importance of technological innovation R&D expenditures to policymakers to fully reap its benefits. We propose that Washington incentivize potential investors to depend more on climate finance and clean energy, which will also ramp up investment in renewable energy-related markets. The role of technological progress could help to channel its importance in the production process. The impact of R&D expenditures shall be channeled to the production process and towards encouraging green energy and innovation. The study is limited to the USA, and its implications cannot be generalized to other economies. Therefore, future studies may focus on other economic data to test its implications for policymakers in other countries.

## Data Availability

The data that support the findings of this study are openly available on request.
